# African swine fever virus pA104R protein acts as a suppressor of type I interferon signaling

**DOI:** 10.3389/fmicb.2023.1169699

**Published:** 2023-04-06

**Authors:** Qichao Chen, Liang Li, Shibang Guo, Zhankui Liu, Lixinjie Liu, Chen Tan, Huanchun Chen, Xiangru Wang

**Affiliations:** ^1^State Key Laboratory of Agricultural Microbiology, College of Veterinary Medicine, Huazhong Agricultural University, Wuhan, China; ^2^Key Laboratory of Preventive Veterinary Medicine in Hubei Province, The Cooperative Innovation Center for Sustainable Pig Production, Wuhan, China; ^3^Key Laboratory of Prevention & Control for African Swine Fever and Other Major Pig Diseases, Ministry of Agriculture and Rural Affairs, Wuhan, China; ^4^International Research Center for Animal Disease, Ministry of Science and Technology of the People’s Republic of China, Wuhan, China

**Keywords:** African swine fever virus, pA104R, type I IFN signaling, STAT1 phosphorylation, immune evasion

## Abstract

This study evaluates the role of the late viral protein, pA104R, in African swine fever virus immunosuppression. ASFV-encoded pA104R is a putative histone-like protein that is highly conserved throughout different virulent and non-virulent isolates. Previous studies have demonstrated that pA104R plays a vital role in the ASFV replication cycle and is a potential target for antiviral therapy. Here, we demonstrated that pA104R is a potent antagonist of type I interferon signaling. IFN-stimulated response element activity and subsequent transcription of co-transfected and endogenous interferon-stimulated genes were attenuated by pA104R treatment in HEK-293 T cells. Immunoprecipitation assay and reciprocal pull-down showed that pA104R does not interact directly with STAT1, STAT2, or IRF9. However, pA104R could inhibit IFN signaling by attenuating STAT1 phosphorylation, and we identified the critical amino acid residues (R/H69,72 and K/R92,94,97) involved through the targeted mutation functional assays. Although pA104R is a histone-like protein localized to the nucleus, it did not inhibit IFN signaling through its DNA-binding capacity. In addition, activation of the ISRE promoter by IRF9-Stat2(TA), a STAT1-independent pathway, was inhibited by pA104R. Further results revealed that both the transcriptional activation and recruitment of transcriptional stimulators by interferon-stimulated gene factor 3 were not impaired. Although we failed to determine a mechanism for pA104R-mediated IFN signaling inhibition other than attenuating the phosphorylation of STAT1, these results might imply a possible involvement of epigenetic modification by ASFV pA104R. Taken together, these findings support that pA104R is an antagonist of type I interferon signaling, which may interfere with multiple signaling pathways.

## 1. Introduction

African swine fever virus (ASFV), the causative agent of the African swine fever (ASF) epidemic, is an enveloped complex double-stranded DNA arbovirus (Jia et al., 2017; Alonso et al., 2018; Gaudreault et al., 2020). The ASFV genome varies in length, ranging from 170 to 193 kb, and encodes 150 to 167 proteins that are used not only for viral replication but also for evasion of host defenses (Sánchez-Vizcaíno et al., 2015; Simões et al., 2019). To date, approximately half of the ASFV genes have not been defined as having any known or predictable functions (Dixon et al., 2017; Alejo et al., 2018). Morphologically, the ASFV virion is a symmetrical icosahedral particle with a diameter of approximately 200 nm and has a complex multi-enveloped structure consisting of a nucleoid, core-shell, inner lipid envelope, protein capsid, and external envelope budding through the plasma membrane (Salas and Andrés, 2013; Wang et al., 2019). ASF is considered one of the most devastating and contagious diseases in swine, manifesting symptoms of viral hemorrhagic fever that result in high morbidity and mortality in domestic swine and wild boars worldwide (Costard et al., 2013; Sánchez-Vizcaíno et al., 2015; Quembo et al., 2018). The World Organization for Animal Health (WOAH) received notifications of outbreaks from 74 countries between 2015 and 2022, indicating a marked increase in ASF outbreaks worldwide (Wu et al., 2020). The relatively rapid spread of this disease, despite concerted containment efforts, is possibly due to the increased transborder movement of pigs and pork products resulting from the growth of pork consumption and international trade. Due to its approximately 100% mortality rate in naive herds and widespread distribution, ASF has caused substantial economic losses for the pig industry and seriously threatens ecological security (Sánchez-Cordón et al., 2018; Zhou et al., 2018; Sánchez et al., 2019). Without available vaccine or treatment, the only control measures are strict quarantine and biosecurity procedures (Sánchez-Cordón et al., 2018). However, massive culling campaigns have only exacerbated the socio-economic impact on global trade and people’s livelihoods.

There are still many gaps in the knowledge regarding the pathological mechanisms of ASFV, and no safe and effective commercial vaccine against the virus has been developed (Dixon et al., 2017; Alejo et al., 2018; Sánchez-Cordón et al., 2018). Therefore, the identification and characterization of ASFV virulence and the mechanisms used by its viral proteins to counter and evade immune responses are essential to develop a viable vaccine and stall the spread of this highly problematic disease. ASFV mainly targets macrophages, where it modulates cell function to replicate in the cytoplasm of the infected cells (Sánchez-Cordón et al., 2018). ASFV devotes considerable coding capacity to genes that support virus proliferation and evasion of host defenses (Randall and Goodbourn, 2008; Reis et al., 2017). The host has multiple levels of antiviral protection, of which innate immunity is the first line of defense. It is well known that viral nucleic acids serve as potent pathogen-associated molecular patterns (PAMPs) and are preferentially recognized by DNA sensors that trigger downstream signaling to elicit innate immune responses, leading to the activation of transcription factors that mediate interferon (IFN) production (Cai et al., 2014; Huang et al., 2018). IFNs are important cytokines in the innate and adaptive immune systems, especially type I IFNs (IFN-α/β), which are produced ubiquitously by virus-infected cells and play a central role in combating viral infections (Goodbourn and Randall, 2009). The secreted IFN-I binds to its cognate receptor subunits (IFNAR1 and IFNAR2) on infected and neighboring cells, activating the Janus kinase signal transducer and activator of the transcription (JAK–STAT) pathway. Once the central mediators STAT1 and STAT2 are activated, these phosphorylated proteins form heterodimers that translocate into the nucleus and associate with IFN regulatory factor 9 (IRF9), assembling into the heterotrimeric transcription factor complex known as interferon-stimulated gene factor 3 (ISGF3) (Platanias, 2005; Stark and Darnell, 2012; Wang et al., 2017). ISGF3 is recruited to the IFN-stimulated response element (ISRE) and enhances the transcription of interferon-stimulated genes (ISGs) that possess ISRE in their promoter regions (Hoffmann et al., 2015), creating a potent antiviral state in cells and limiting viral reproduction (Platanias, 2005; Stark and Darnell, 2012; Wang et al., 2017).

To effectively infect and replicate, viruses have, in turn, devised various strategies to combat the production of ISGs through the inhibition of the JAK/STAT pathway activated by IFN and antagonizing of host antiviral defenses (Katze et al., 2002; Goodbourn and Randall, 2009; Schulz and Mossman, 2016; García-Sastre, 2017; Nan et al., 2017). Recently, it has been reported that ASFV targets crucial molecules in IFN signaling to counteract immune responses, but which viral proteins are responsible remains unclear (Portugal et al., 2018; Wang J. et al., 2020; Wang T. et al., 2020; Riera et al., 2021; Cui et al., 2022). pA104R is a putative histone-like protein encoded by ASFV, analogous to eukaryotic histones (Neilan et al., 1993), that shares structure and sequence homology with members of the HU/IHF family (Bonnefoy and Rouvière-Yaniv, 1991; Borca et al., 1996; Browning et al., 2010), which are primary DNA-packaging proteins in prokaryotes (Bonnefoy and Rouvière-Yaniv, 1991; Swinger and Rice, 2004). Similarly, as the only histone-like protein encoded by a eukaryotic virus (Borca et al., 1996), pA104R also binds to single-or double-stranded DNA over a wide range of conditions in an ATP-independent manner (Frouco et al., 2017). Previous studies have demonstrated that pA104R displays DNA supercoiling activity when combined with ASFV topoisomerase II (Freitas et al., 2019); neither exhibits this activity alone, suggesting the two proteins cooperate in this process. Accumulating evidence suggests that a late viral protein colocalizes with the cell nucleus (Alejo et al., 2018) that requires pA104R for viral DNA replication, repair, recombination, and transcription (Frouco et al., 2017). pA104R might also be involved in nucleoid compaction and progeny assembly processes (Neilan et al., 1993) or, alternatively, act as a transcription factor modulating viral gene expression (Neilan et al., 1993; Borca et al., 1996; Frouco et al., 2017). However, the biological functions of the pA104R protein are largely unknown. In the present study, we revealed that the ASFV pA104R protein antagonizes the IFN-I-triggered signaling pathway by attenuating the phosphorylation of STAT1. Additionally, we presumed epigenetic modifications that may be mediated through the same signaling and have a role in pA104R pathogenicity. Our findings demonstrate a previously unidentified function of pA104R in ASFV evasion of host innate immunity. These findings contribute significantly to the theoretical basis for designing an effective ASFV vaccine.

## 2. Materials and methods

### 2.1. Cells and plasmids

Human embryonic kidney 293 T (HEK-293 T) cells (ATCC CRL-3216, Manassas, VA) were cultured in Dulbecco’s Modified Eagle Medium (DMEM) supplemented with 10% (v/v) fetal bovine serum (FBS) and maintained in a humidified incubator with 5% CO_2_ at 37°C. The expression vectors IRF9-Stat2(TA) and GAL4-Stat2(TA) were constructed by inserting a PCR-amplified STAT2 transactivation domain into IRF9 or GAL4-DBD plasmids (Sadowski and Ptashne, 1989). STAT1, STAT2, and IRF9 were cloned into the plasmids indicated. The ASFV gene A104R was amplified from ASFV CN/SD/2019 genomic DNA and cloned into pCAGGS-HA with an N-terminal HA tag. ASFV pA104R residue mutations were generated by site-directed mutagenesis using the wild-type plasmid pCAGGS-HA-A104R as a template. All constructed plasmids were confirmed using DNA sequencing.

### 2.2. Antibodies and reagents

The STAT1 (9172), STAT2 (72604), phosphor-STAT1 (9649), phosphor-STAT2 (88410), and IRF9 (76684) antibodies were purchased from Cell Signaling Technology (Danvers, MA). β-Actin (66009-1-Ig), GFP-tag (66002-1-Ig), Flag-tag (20543-1-AP), and HA-tag (51064-2-AP) antibodies were purchased from Proteintech (Chicago, IL). Recombinant human IFN-α (CYT-204) was purchased from ProSpec (Ness Ziona, Israel). The jetPRIME® transfection reagent was purchased from Polyplus-transfection® SA (Illkirch, France). Streptavidin magnetic beads (HY-K0208) and protein A/G magnetic beads (HY-K0202) were purchased from MedChemExpress (Monmouth Junction, NJ).

### 2.3. Dual-luciferase reporter assay

HEK-293 T cells were seeded in 24-well plates and transfected with the indicated expression plasmids or empty vector control with 50 ng pISRE-Luc or pGAL4-UAS-Luc plasmid (Firefly) and 10 ng pRL-TK plasmid (Renilla) as an internal control. A constant level of total DNA was maintained by adding an empty vector. At 24 h post-transfection, the cells were stimulated with DMEM or IFN-α (1,000 U/mL) for 12 h, and whole-cell lysates were then collected to measure luciferase activity using the Dual-Luciferase® Reporter Assay System (Promega, Madison, WI), according to the manufacturer’s instructions. Relative luciferase activity was normalized by the ratio of firefly luciferase activity to Renilla luciferase activity.

### 2.4. RNA extraction and quantitative real-time PCR

Briefly, total RNA was extracted using TRIzol reagent (Thermo Fisher Scientific, Waltham, MA) following the manufacturer’s instructions. Then, real-time PCR was performed using a MonAmpTM SYBR® Green qPCR Mixture (Monad Biotech Co., Ltd., Wuhan, China) and a QuantStudio 3 PCR system (Thermo Fisher Scientific, Waltham, MA). Sample data were normalized to GAPDH mRNA levels. Experiments were performed in biological triplicate and conducted three times. The specific primers used for RT-qPCR assays have been described previously (Li et al., 2022).

### 2.5. Co-immunoprecipitation and immunoblotting assays

Cells were lysed and harvested using cell lysis buffer (Beyotime, Shanghai, China) supplemented with Protease/Phosphatase Inhibitor Cocktail (Cell Signaling Technology, Danvers, MA). The protein concentrations in the supernatants were measured using a BCA protein assay kit (Biosharp, Anhui, China). For the co-immunoprecipitation experiments, equal amounts of cell lysates were incubated by rotation with the indicated antibody or control IgG at 4°C overnight. Subsequently, the samples were incubated with protein A/G magnetic beads for 4 h, washed five times with lysis buffer, and boiled in sodium dodecyl sulfate (SDS) loading buffer. The precipitates were subjected to SDS-PAGE and subsequent immunoblotting using the indicated antibodies, which were consistent with those described previously (Li et al., 2022).

### 2.6. Biotinylated-DNA immunoprecipitation

The probe used in DNA immunoprecipitation was obtained by annealing biotin-labeled ISRE or control oligonucleotides and their complement. The biotin probe was incubated with nuclear extracts for 4 h at 4°C. Samples were then pulled down using streptavidin magnetic beads at 4°C for 2 h. After washing, ISRE-binding proteins were immunoblotted using the indicated antibodies. The biotin-labeled control oligonucleotide was an ISRE sequence in which the core region was replaced by GFP. Nuclear extracts were prepared from cells treated with IFN-α after transfection with the plasmids described previously in the “cells and plasmids” subsection of our methods (de Lucas et al., 2005).

### 2.7. Statistical analysis

Statistical analysis was performed using GraphPad Prism software (version 7.0, GraphPad Software, La Jolla, CA, United States) to perform a Student’s *t*-test or one-way analysis of variance (ANOVA) on at least three independent replicates. For each test, *p* values <0.05 were considered statistically significant (* *p* < 0.05, ** *p* < 0.01, and *** *p* < 0.001). Data are presented as the mean ± standard error of the mean (mean ± SEM) from at least three replicates.

## 3. Results

### 3.1. ASFV pA104R antagonizes type I IFN signaling

**Figure 1:**
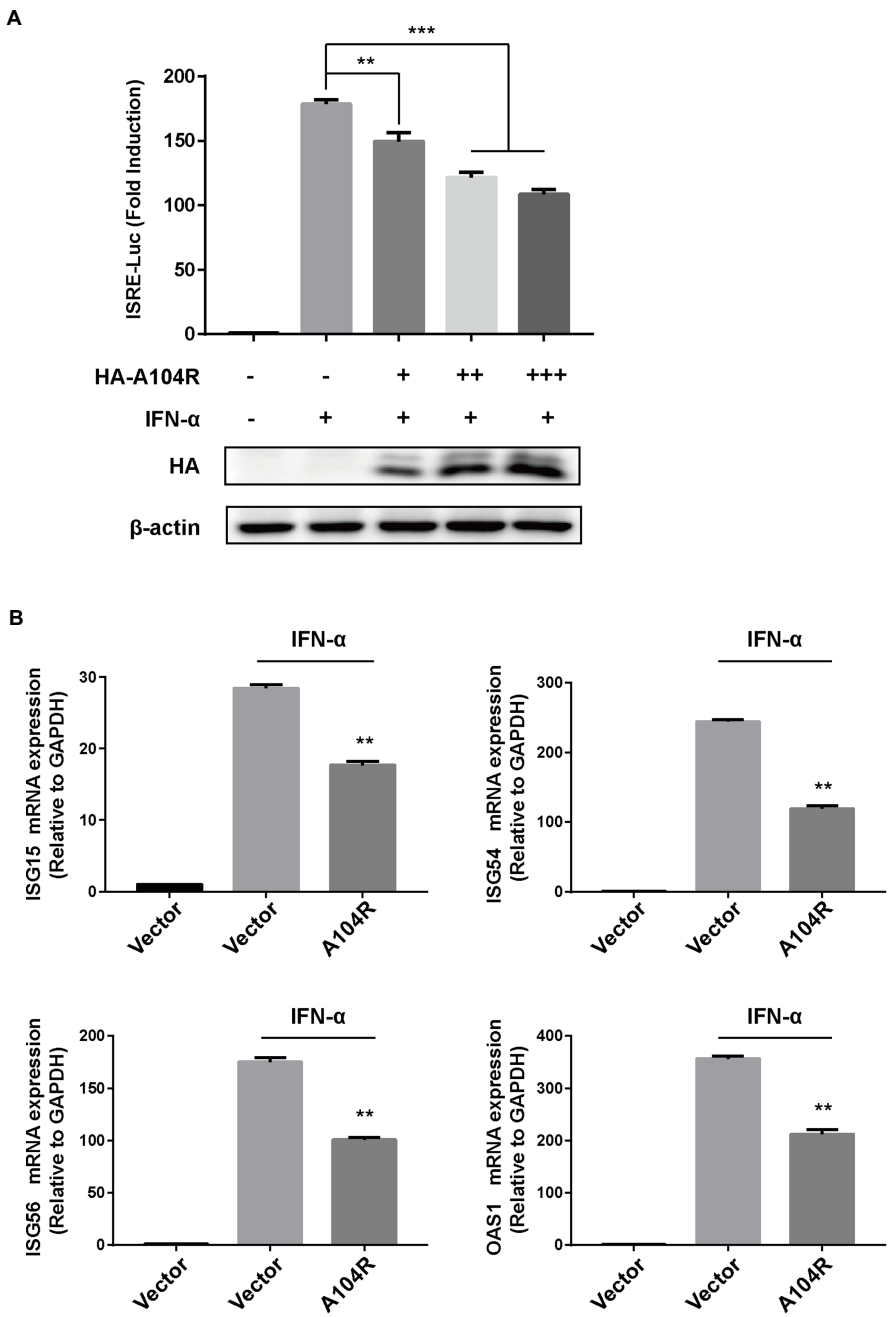
ASFV pA104R inhibits type I IFN signaling. **(A)** HEK-293T cells cultured in 24-well plates were transfected with various concentrations of pA104R plasmids (0, 0.2, 0.4, 0.6 μg/well), or empty vector along with pISRE-Luc and pRL-TK plasmids. After 24 h, cells were treated with 1,000 U/mL IFN-α for 12 h, and luciferase assays were then performed. The protein levels of pA104R were evaluated using immunoblotting analysis. **(B)** HEK-293T cells were transfected with pA104R, or empty vector. At 24 h post-transfection, cells were treated with 1,000 U/mL IFN-α for 8 h, and the mRNA levels of ISGs were analyzed by RT-qPCR. Statistical analysis was performed using Student’s t-test. All experiments were performed in triplicate, and the data represent mean values ± SEM for triplicates. ** *p* < 0.01; *** *p* < 0.001.

It is well known that IFN-I initiates a series of signaling cascades, inducing the expression of many ISGs. The resulting gene products collaboratively fight viral infections and contribute to the implementation of adaptive immune responses (Platanias, 2005). ASFV is an immunosuppressive virus that encodes various proteins to counter key signal transduction processes in innate immunity (Zheng et al., 2022). However, the functions of many of these proteins remain unclear. To determine whether pA104R could inhibit signaling downstream of IFN-I, we assessed its effect on the expression of an ISRE-dependent luciferase reporter gene. HEK-293 T cells were co-transfected with pISRE-Luc (containing a consensus sequence for the IFN-stimulated response element of the ISG promoter), pRL-TK (internal control plasmid), and different concentrations of pA104R vectors. Treatment of HEK-293 T cells with IFN-α led to the induction of luciferase expression, and we demonstrated that pA104R attenuated ISRE promoter activity in a dose-dependent manner (Figure 1A). To confirm the ability of pA104R to inhibit IFN-I signaling, the effect of pA104R on the induced transcription of endogenous ISGs was assessed. IFN-α treatment of cells resulted in the induction of well-characterized ISGs, including IFN-stimulated gene 15 (ISG15), ISG54, ISG56, and 2′-5′-oligoadenylate synthetase 1 (OAS1). However, the presence of pA104R significantly inhibited the induction of ISGs expression compared to that in the controls (Figure 1B). These results confirm the antagonistic role of ASFV pA104R in type I IFN signaling.

### 3.2. pA104R attenuates IFN-α-mediated phosphorylation of STAT1

**Figure 2:**
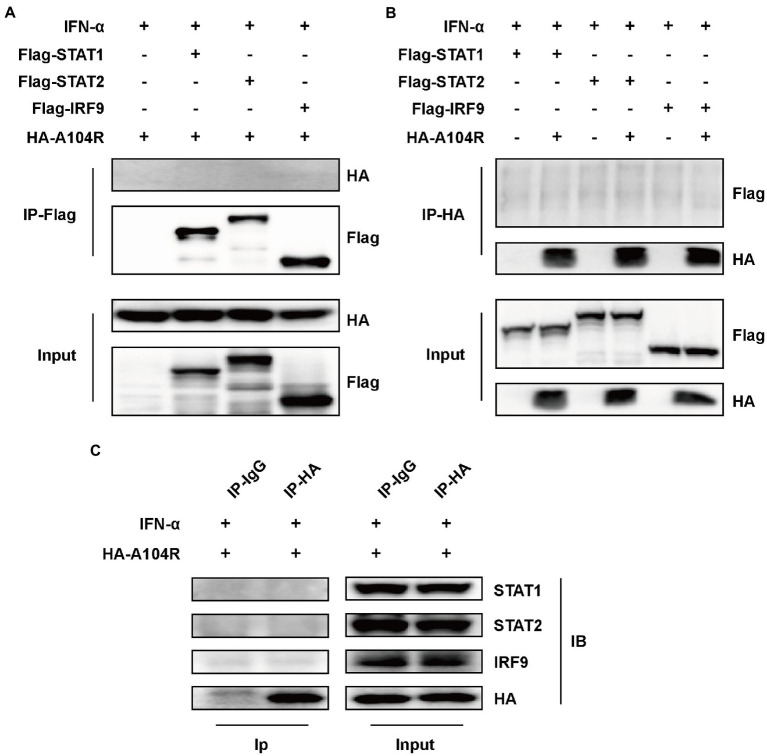
ASFV pA104R inhibits ISGF3-induced ISRE promoter activity and attenuates the phosphorylation of STAT1. **(A)** HEK-293 T cells were transfected with various concentrations of pA104R plasmids, along with ISGF3 complex (STAT1, STAT2 and IRF9) and pISRE-Luc and pRL-TK plasmids. After 30 h, a luciferase assay was performed. Data are shown as means ± SEM from three independent experiments. Statistical analysis was performed by one-way ANOVA. * *p* < 0.05; ** *p* < 0.01; *** *p* < 0.001. **(B)** HEK-293 T cells were transfected with pA104R or empty vector. After 24 h, cells were treated with 1,000 U/ml IFN-α for 2 h. The levels of total or phosphorylated STAT1, STAT2, and IRF9 were detected by immunoblotting analysis.

Considering the importance of the transcription factor complex ISGF3 in type I IFN signaling, we investigated whether the overexpression of pA104R inhibited ISGF3-mediated signaling. As shown, activation of the ISRE promoter was markedly induced by co-expression of the components of ISGF3 (STAT1, STAT2, and IRF9) compared with the empty plasmid control. However, pA104R significantly inhibited ISGF3-induced activation of the ISRE promoter in a dose-dependent manner (Figure 2A), suggesting that the pA104R exerts its inhibitory effect on IFN-I signaling either during or immediately following the assembly of ISGF3 components.

Subsequently, we examined the endogenous protein levels and phosphorylation status of STAT1, STAT2, and IRF9 in HEK-293 T cells expressing pA104R following IFN-α treatment. The results demonstrated that no reduction was observed in the endogenous protein levels of the ISGF3 components (STAT1, STAT2, and IRF9) in the presence or absence of pA104R. However, the inhibition of STAT1 phosphorylation was observed in the presence of pA104R. In contrast, the phosphorylation of STAT2 was maintained at a steady-state level with or without pA104R (Figure 2B). These observations indicate that pA104R prevents IFN-α-induced phosphorylation of STAT1.

### 3.3. pA104R does not interact with STAT1, STAT2 or IRF9

**Figure 3:**
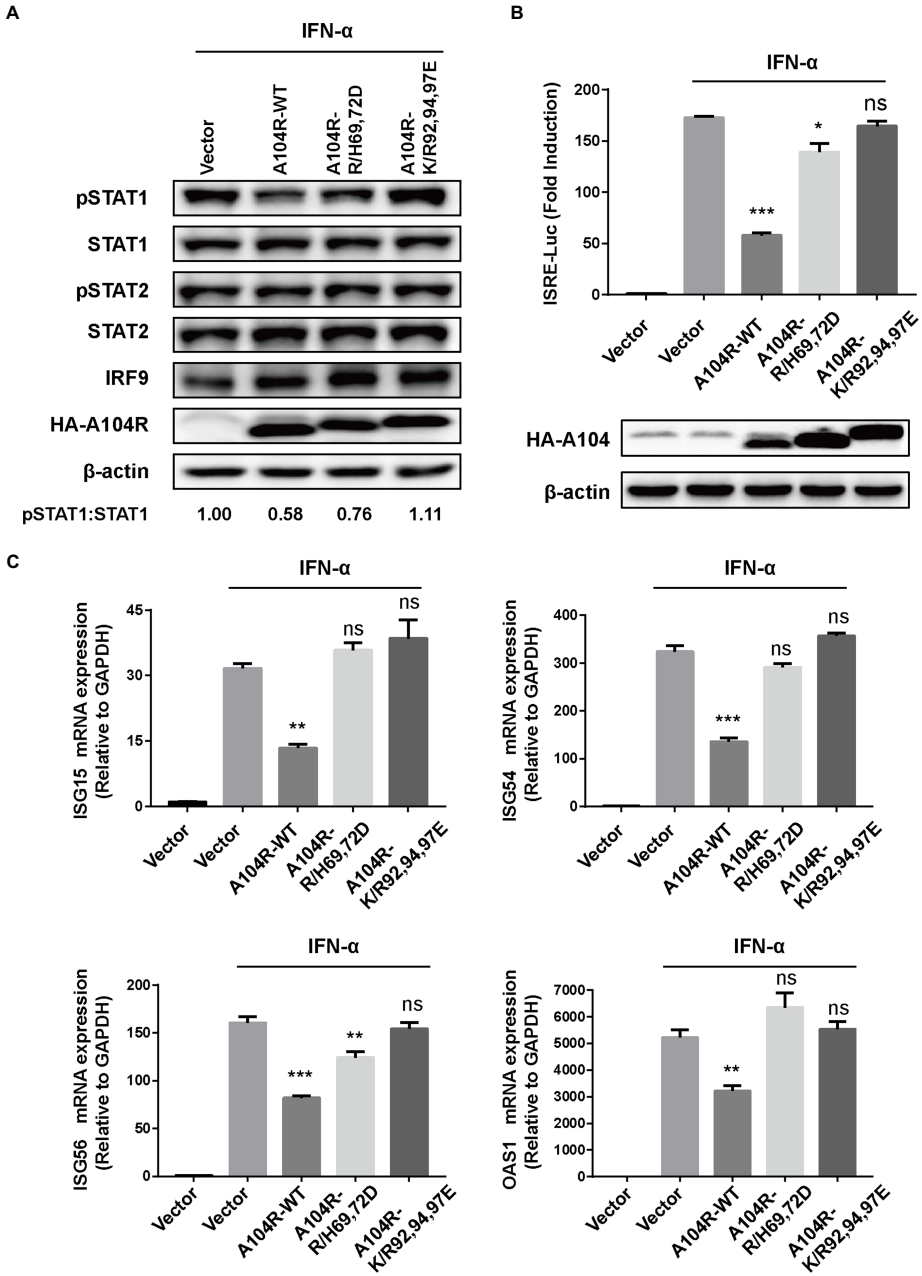
ASFV pA104R does not interact with STAT1, STAT2, and IRF9. HEK-293 T cells were transfected with pA104R alone **(C)** or co-transfected with STAT1, STAT2, and IRF9 **(A**,**B)**. After 24 h, cells were treated with 1,000 U/mL IFN-α for 8 h. Cell lysates were prepared and subjected to immunoprecipitation analysis. The whole-cell lysates and immunoprecipitation complexes were analyzed by immunoblotting with the indicated antibodies.

Several viral proteins can interact with components of the ISGF3 complex to inhibit IFN-I signaling (Zhang et al., 2012, 2015; Oda et al., 2015). Therefore, we speculated that the observed inhibition of STAT1 phosphorylation resulted from interactions of pA104R with STAT1 or other components of ISGF3. To this end, an immunoprecipitation assay using FLAG-tagged ISGF3 was performed. As shown in Figures 3A,B, the reciprocal pull-down of pA104R and ISGF3 components confirmed no interaction between them. To exclude the possibility of interaction between pA104R and some component of ISGF3 occurring at endogenous protein levels, co-precipitated overexpression of pA104R (or empty vector control) with endogenous STAT1, STAT2 or IRF9 was followed by immunoblot assays. Western blot analysis showed that pA104R did not interact with endogenous STAT1, STAT2, or IRF9 (Figure 3C). Collectively, these data indicate the inhibitory effect of pA104R on IFN-I signaling was not caused by direct interaction with the ISGF3 complex or its constituents.

### 3.4. pA104R-mediated inhibition of IFN-I signaling is associated with its DNA-binding catalytic residues

**Figure 4:**
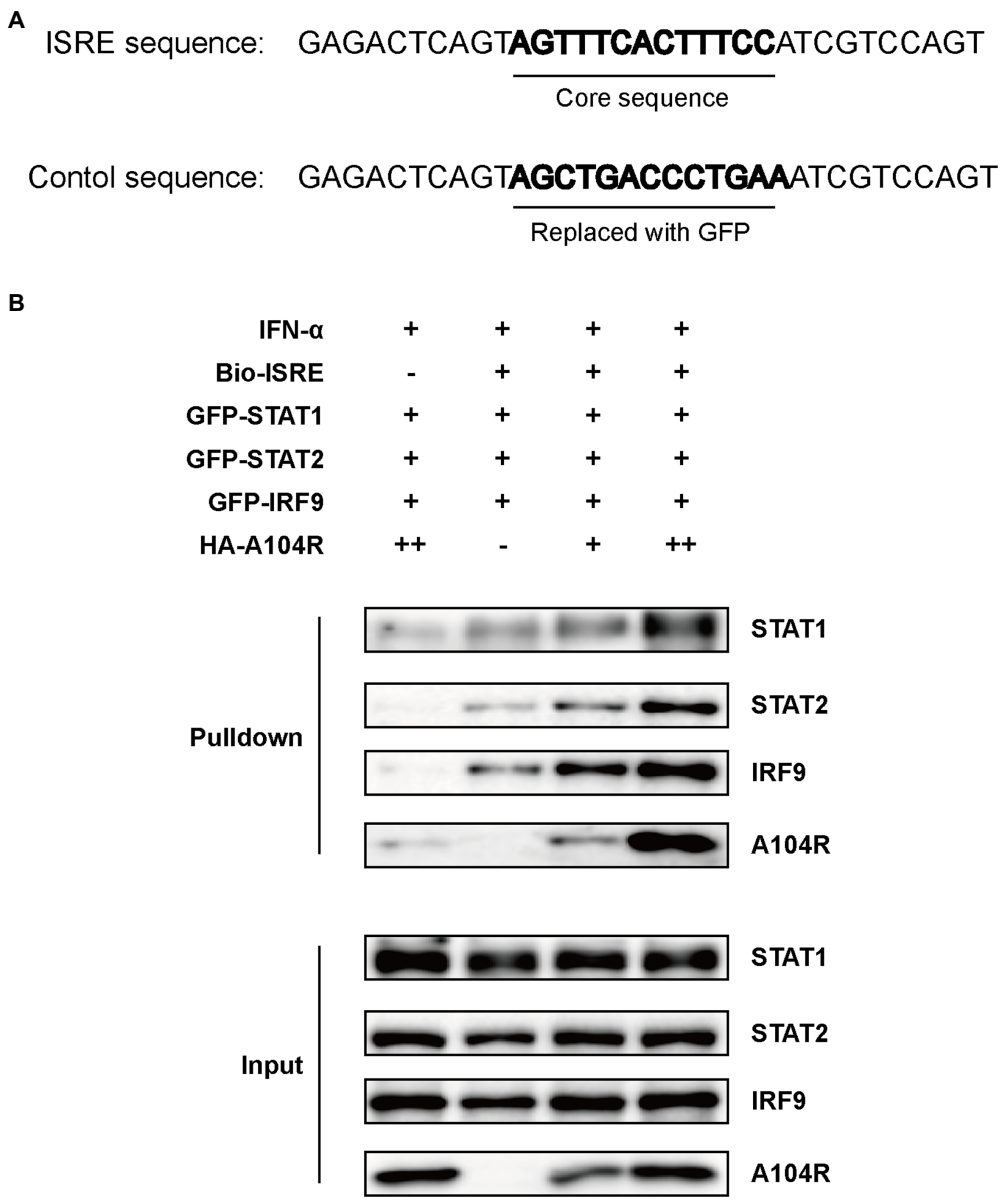
ASFV pA104R-Mediated Inhibition of IFN-I signaling is associated with its DNA-binding catalytic residues. Cells were transfected with pA104R or its DNA-binding activity-defective mutants. After 24 h, cells were treated with 1,000 U/mL IFN-α for 2 h **(A)**, 8 h **(B)**, or 12 h **(C)**. Cell lysates were used for immunoblotting analysis with the indicated antibodies **(A)**, used for luciferase assay **(B)**, or used for analyzing the mRNA levels of ISGs **(C)**. Data are shown as means ± SEM from three independent experiments. Statistical analysis was performed by one-way ANOVA. * *p* < 0.05; ** *p* < 0.01; *** *p* < 0.001.

Key catalytic residues that mediate the DNA-binding activity of pA104R and their essential roles in the ASFV replication cycle have been characterized (Liu et al., 2020). Therefore, we hypothesized that these DNA-binding catalytic residues play a role in disturbing type I IFN signaling. Based on their structural properties and the known residue requirements, five catalytic residues (Arg69, His72, Lys92, Arg94, and Lys97) were identified to be directly involved in DNA-binding activity (Frouco et al., 2017; Liu et al., 2020). We thus determined whether mutation of these residues would negate the ability of pA104R to inhibit STAT1 phosphorylation. Two residue mutations, R/H69,72D and K/R92,94,97E, were introduced into pA104R and the corresponding eukaryotic expression plasmids. As we speculated, immunoblot analysis suggested that the mutant protein attenuated the reduction in phosphorylated STAT1 compared to wild-type pA104R (Figure 4A).

In addition, we found that mutations in the pA104R DNA-binding catalytic residues reversed the inhibition of IFN-α-induced ISRE promoter activity (Figure 4B). Furthermore, we examined the ability of the pA104R mutant to inhibit ISG expression. As shown in Figure 4C, each mutation (R/H69,72D and K/R92,94,97E) also attenuated the pA104R-mediated inhibition of IFN-α-induced transcription of ISGs. These results suggest that the pA104R-mediated inhibition of type I IFN signaling is closely associated with these DNA-binding catalytic residues.

### 3.5. pA104R does not prevent the association of ISGF3 with DNA

**Figure 5:**
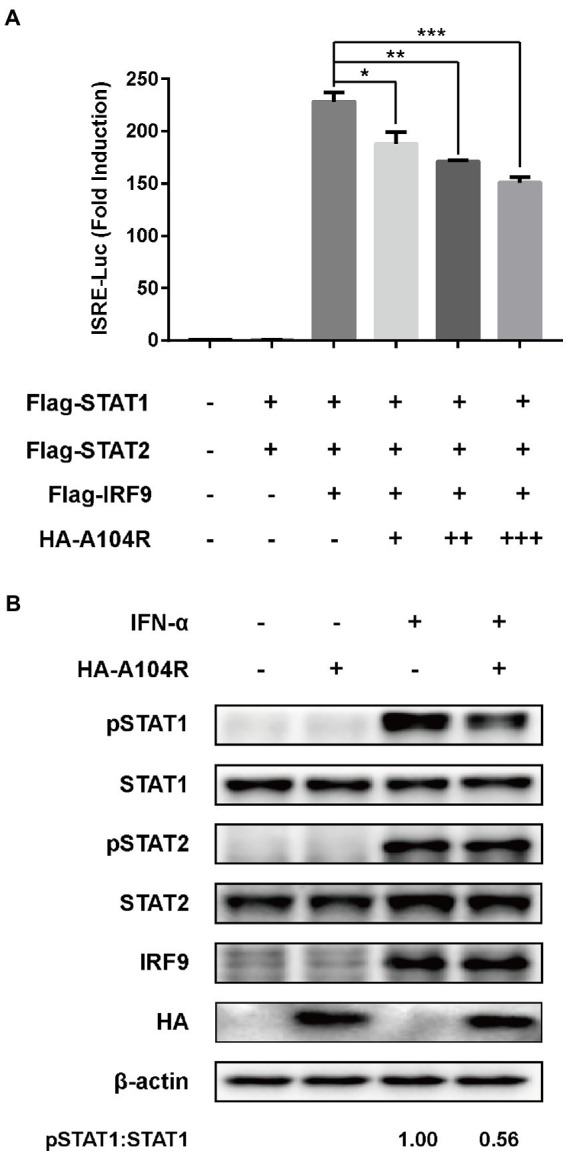
ASFV pA104R does not prevent the association of ISGF3 with promoter. **(A)** The ISRE DNA and control oligonucleotides used for DNA pull-down. **(B)** HEK-293T cells were co-transfected with ISGF3 complex (STAT1, STAT2, and IRF9), along with pA104R or empty control. After 24 h post-transfection, cells were treated with 1,000 U/mL IFN-α for 12 h. Nuclear extracts were incubated with a Biotin-labeled ISRE or control probe and subjected to pull-down analysis with streptavidin magnetic beads. The whole-cell lysates and pull-down complexes were analyzed by immunoblotting with the indicated antibodies.

Previous studies have characterized pA104R as a histidine-like protein with DNA-binding activity and a higher affinity for dsDNA than for ssDNA (Frouco et al., 2017). Considering that the downstream effects of IFN-I require the binding of ISGF3 to ISREs (Kessler et al., 1990), we hypothesized that pA104R may act by directly interacting with the promoters of ISGs, except for its inhibiting the phosphorylation of STAT1. To determine whether pA104R inhibited IFN-α signaling by preventing the binding of the ISGF3 complex to ISREs in ISG promoters, we performed a DNA pull-down assay to determine whether pA104R disrupted ISGF3 binding to ISREs. HEK-293 T cell were co-transfected with pA104R and green fluorescent protein (GFP)-tagged ISGF3 components (STAT1, STAT2, and IRF9). A biotin-labeled ISRE probe, optimized previously for ISGF3 binding (Biotin-ISRE), or a control biotin-labeled sequence that replaced the ISRE core region with a sequence from GFP (Figure 5A) were incubated with nuclear extract followed by applying the streptavidin beads to immuno-precipitate the biotinylated DNA probe and associated proteins (Schmid et al., 2010; Qin et al., 2019). ISGF3 components were pulled down by biotin-labeled ISRE but not by biotin-labeled control (Figure 5B; Shen et al., 2020). These findings agree with a previous report that pA104R can interact with the biotin-labeled ISRE and control (Frouco et al., 2017). Unexpectedly, the expression of pA104R did not inhibit the binding of ISGF3 components to biotin-labeled ISRE, suggesting that the DNA-binding activity of pA104R does not govern its ability to block ISGF3-induced, ISRE promoter-associated gene expression.

### 3.6. pA104R repression of IFN signaling may occur through epigenetic transcriptional regulation

**Figure 6:**
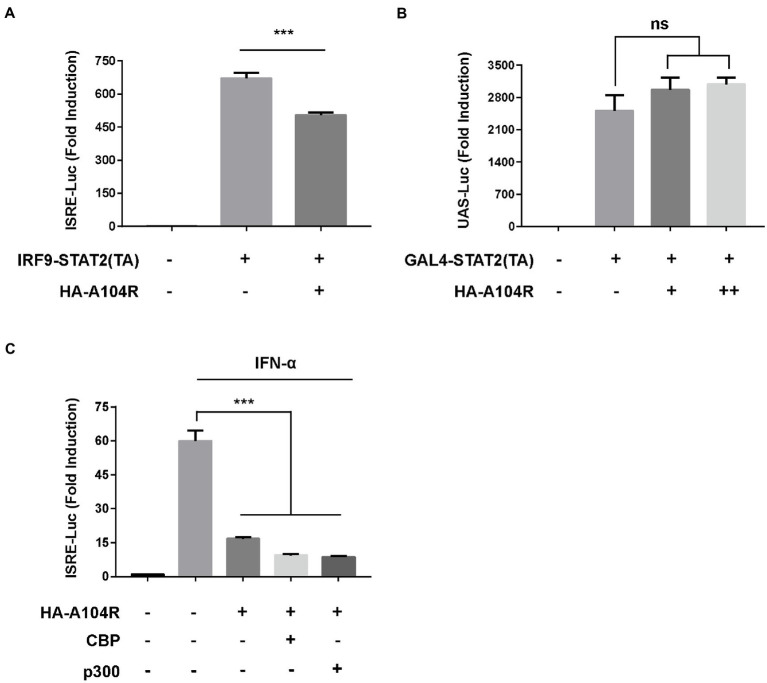
ASFV pA104R does not exert repressive effects on trans-activation domains and transcriptional co-stimulatory factors. **(A)** HEK-293 T cells were co-transfected with pA104R and IRF9-Stat2(TA) **(A)** or GAL4-Stat2(TA) **(B)**, along with pRL-TK and pISRE-Luc **(A)** or pGAL4-UAS-Luc **(B)** plasmids. After 30 h, a luciferase assay was performed. **(C)** HEK-293 T Cells were co-transfected with pA104R and CBP or p300 along with pISRE-Luc and pRL-TK plasmids. After 24 h post-transfection, cells were treated with 1,000 U/mL IFN-α for 12 h, followed by luciferase assays. Data are shown as means ± SEM from three independent experiments. Statistical analysis was performed by one-way ANOVA. ^***^ *p* < 0.001.

We further investigated whether pA104R played a repressive role downstream of transcription factor binding to promoter regions. The plasmid IRF9-Stat2(TA), a chimera containing the full length of IRF9 and the transactivation domain of STAT2, was used to verify the role of pA104R. As shown in Figure 6A, IRF9-Stat2 (TA) expression significantly activated ISRE promoter activity. Consistently, activation of the ISRE promoter by IRF9-Stat2(TA) was inhibited by the presence of pA104R. Interestingly, this result suggests that the inhibitory effects of pA104R on IFN signaling are STAT1-independent. It is generally accepted that the C-terminus of STAT2 serves as a transcriptional activation domain (TA), which is essential for ISGF3-driven transcription (Qureshi et al., 1996). To further clarify this, we constructed the pGAL4-Stat2(TA) plasmid (Zhang et al., 2008) and examined whether pA104R inhibits the transactivation function of STAT2. The expression of the reporter gene from the pGAL4-UAS-Luc plasmid in HEK-293 T cells was markedly activated by co-transfection with pGAL4-Stat2(TA). However, no noticeable reduction was observed in the transactivation activity driven by pGAL4-Stat2(TA) in the presence or absence of pA104R (Figure 6B), demonstrating that pA104R-mediated inhibition was not due to impairment of the general transcription machinery. It has been demonstrated that STATs and STAT-containing ISGF3 function in transcriptional activation partly through the recruitment of CBP/p300, a ubiquitously expressed global transcriptional coactivator with histone acetyltransferase (HAT) activity (Bhattacharya et al., 1996; Zhang et al., 1998, 2008). As shown in Figure 6C, exogenously expressed CBP/p300 could not restore the reduced ISRE promoter activity by pA104R in response to IFN-α, indicating that pA104R does not exert an inhibitory effect by targeting CBP/p300. Considering all aspects of these data, we speculate that pA104R functions as an antagonist of IFN signaling and may also have epigenetic modification effects involved in the ISG transcription process, which requires further investigations.

## 4. Discussion

The continuous evolutionary arms race dynamically shapes the diverse survival strategies of viruses and their hosts. Innate immunity serves as the first line of defense against invading pathogens, inducing the expression of hundreds of ISGs *via* the IFN signaling pathway to establish an antiviral state. In turn, viruses have evolved various strategies in concert, targeting different steps in the IFN signaling pathways to subvert the antiviral response for their survival (Stetson and Medzhitov, 2006; Hervas-Stubbs et al., 2011; Riera Romo et al., 2016). The primary target cells of ASFV replication are macrophages, which specialize in innate immune responses to pathogens. Therefore, to overcome barriers to replication in macrophages, ASFV devotes considerable coding capacity to genes that help the virus survive and inhibit host defense pathways (Reis et al., 2017; Portugal et al., 2018). Recently, increasing evidence suggests that several proteins encoded by ASFV can circumvent innate immunity by targeting IFN signaling through different mechanisms (Correia et al., 2013; Wang et al., 2018; García-Belmonte et al., 2019; Zhuo et al., 2020; Li et al., 2021).

ASFV pA104R is a histone-like protein mainly translated during the late phase of infection. Its nuclear localization supports that pA104R primarily participates in viral DNA replication, transcription, and genome packaging (Simões et al., 2015a). However, the role of pA104R in negating host innate immunity strategies remains to be explored. In the present study, we found that ASFV pA104R inhibited IFN-α-induced ISG production and ISRE promoter activation and identified pA104R as a potential suppressor of innate immunity (Figure 1). ISGF3 is well characterized as a central mediator of type I IFN signaling (Chen et al., 2017). In response to IFN-α stimulation, STAT1, STAT2, and IRF9 assemble to form the transcription factor complex ISGF3 (Qureshi et al., 1995; Wang D. et al., 2019), which is transported to activate ISRE (Wang D. et al., 2019; Zhang et al., 2022). We have demonstrated that pA104R inhibited ISGF3-mediated ISRE luciferase signaling (Figure 2A), suggesting that pA104R is a suppressor of IFN signaling that likely targets ISGF3 itself or a related downstream process. Previous studies have shown that many viral proteins can subvert the IFN-I signaling pathway by targeting IRF9 or STATs to reduce phosphorylation or stimulate degradation (Palosaari et al., 2003; Fanunza et al., 2021; Feng et al., 2021). Further studies revealed that although pA104R did not interact with any component of ISGF3 (Figure 3), it still attenuated the phosphorylation of STAT1 (Figure 2B). Considering the essential role of the DNA-binding activity of pA104R in ASFV replication (Simões et al., 2015a), we focused on the significance of the corresponding catalytic residues in IFN signaling. As expected, the mutation of the corresponding DNA-binding residues could revert the inhibitory effect of pA104R (Figure 4). Besides, some viruses could directly inhibit ISGF3 binding to DNA (Vidy et al., 2007). Therefore, it is also possible that pA104R recognizes and binds to ISRE to prevent ISGF3 binding. Although the DNA-binding activity of pA104R was observed, the interaction of ISGF3 and DNA was not prevented (Figure 5B). Previous reports have shown that high levels of STAT2 and IRF9 can form heterodimers and contribute to the activation of ISG expression *via* a non-classical pathway (Fink and Grandvaux, 2013). Although it lacks intrinsic transcriptional capacity, IRF9 is a DNA sequence recognition subunit of ISGF3 that is essential for the ISGF3 complex to bind to consensus ISREs (Levy et al., 1989; Martinez-Moczygemba et al., 1997). On the other hand, modular STAT2 provides essential transcriptional activation signals for the induction of target gene transcription (Horvath, 2000). The interaction of IRF9 and the STAT2 transactivation domain can activate antiviral signaling independent of the activity of the ISGF3 complex, indicating an approach for active antiviral responses in the absence of IFN-I stimulation (Kraus et al., 2003). Interestingly, the results in Figure 6A show that pA104R also attenuated IRF9-Stat2(TA)-activated ISRE promoter activity, suggesting there may be a second mechanism by which pA104R inhibits IFN signaling. In addition to the binding of the promoter, the transactivation domain of STAT2 and transcriptional co-stimulator, CREB-binding protein (CBP/p300), are essential for normal IFN signaling (Tang et al., 2007; Zhang et al., 2008; Shen et al., 2020). However, these proteins were not disrupted by pA104R treatment (Figures 6B,C). It is noteworthy that a variety of cellular proteins involved in epigenetic transcriptional regulation have been identified as essential components of ISGF3-driven transcription, such as chromatin remodeling complexes histone and deacetylases, or acetyltransferases (HATs or HDACs) (Bhattacharya et al., 1996; Huang et al., 2002; Liu et al., 2002; Lau et al., 2003; Nusinzon and Horvath, 2003; Cui et al., 2004; Gnatovskiy et al., 2013). A case in point, influenza A virus NS1 and Adenovirus E1A have inhibitory effects downstream of ISGF3 promoter binding through interaction with a complex involved in transcriptional elongation (hPAF1C) and histone ubiquitylating complexes, respectively (Fonseca et al., 2012; Marazzi et al., 2012). Therefore, it is not rare that viruses alter the epigenetic state of host cell chromosomes to control cellular gene expression for their benefit (de Souza et al., 2010; Knipe et al., 2013). It is possible that ASFV can interfere with the epigenetic status of the host chromatin (Simões et al., 2019), and pA104R might participate in the heterochromatinization of the genome (Simões et al., 2015b), which could facilitate viral infection by silencing genes required for immune response (Frouco et al., 2017). In addition to inhibiting STAT1 phosphorylation, pA104R may function as an antagonist of IFN signaling through such epigenetic modifications.

Previously, it was believed that pA104R is an essential viral protein for ASFV replication (Freitas et al., 2019), and we are currently unable to generate a pA104R-defective virus to further evaluate the role of pA104R in IFN signaling. However, the development of a recombinant virus lacking the pA104R gene has recently been reported. These differences may be due to differences in the genomes of different viral isolates. Interestingly, deletion of the pA104R gene in ASFV induces an apparent decrease in virulence (Ramirez-Medina et al., 2022), probably due to the elimination of immunosuppression mediated by pA104R, which also supports our conclusions. However, future studies are needed to assess further the exact consequences of pA104R involvement in epigenetic modifications and to validate these mechanisms using a pA104R-deficient ASFV infection model.

## Data availability statement

The original contributions presented in the study are included in the article/supplementary material, further inquiries can be directed to the corresponding author.

## Author contributions

QC and XW conceived and designed the experiments. QC carried out the experiments and drafted the manuscript. LiaL, SG, ZL, and LixL provided valuable technical assistance for the experiments and analyzed the data. XW revised the manuscript. XW, HC, and CT provided technical and administrative support. All authors contributed to the article and approved the submitted version.

## Funding

This work was funded by the National Natural Science Foundation of China (grant no. 31941005).

## Conflict of interest

The authors declare that the research was conducted in the absence of any commercial or financial relationships that could be construed as a potential conflict of interest.

## Publisher’s note

All claims expressed in this article are solely those of the authors and do not necessarily represent those of their affiliated organizations, or those of the publisher, the editors and the reviewers. Any product that may be evaluated in this article, or claim that may be made by its manufacturer, is not guaranteed or endorsed by the publisher.
